# Monitoring of Chlorophyll Content of Potato in Northern Shaanxi Based on Different Spectral Parameters

**DOI:** 10.3390/plants13101314

**Published:** 2024-05-10

**Authors:** Hongzhao Shi, Xingxing Lu, Tao Sun, Xiaochi Liu, Xiangyang Huang, Zijun Tang, Zhijun Li, Youzhen Xiang, Fucang Zhang, Jingbo Zhen

**Affiliations:** 1 Key Laboratory of Agricultural Soil and Water Engineering in Arid and Semiarid Areas of Ministry of Education, Northwest A&F University, Xianyang 712100, China; shihongzhao7@nwsuaf.edu.cn (H.S.); luxingxing625@163.com (X.L.); 2021050986@nwsuaf.edu.cn (T.S.); 2023055903@nwafu.edu.cn (X.L.); huangxiangyang2023@163.com (X.H.); tangzijun@nwsuaf.edu.cn (Z.T.); zhangfc@nwsuaf.edu.cn (F.Z.); zhenjingbo@nwsuaf.edu.cn (J.Z.); 2 Institute of Water–Saving Agriculture in Arid Areas of China, Northwest A&F University, Xianyang 712100, China; 3 Department of Mechanical Engineering, College of Mechanical and Electrical Engineering, Yangling Vocational & Technical College, Xianyang 712100, China

**Keywords:** potato, hyperspectral, chlorophyll content, machine learning

## Abstract

Leaf chlorophyll content (LCC) is an important physiological index to evaluate the photosynthetic capacity and growth health of crops. In this investigation, the focus was placed on the chlorophyll content per unit of leaf area (LCC_A_) and the chlorophyll content per unit of fresh weight (LCC_W_) during the tuber formation phase of potatoes in Northern Shaanxi. Ground-based hyperspectral data were acquired for this purpose to formulate the vegetation index. The correlation coefficient method was used to obtain the “trilateral” parameters with the best correlation between potato LCC_A_ and LCC_W_, empirical vegetation index, any two-band vegetation index constructed after 0–2 fractional differential transformation (step size 0.5), and the parameters with the highest correlation among the three spectral parameters, which were divided into four combinations as model inputs. The prediction models of potato LCC_A_ and LCC_W_ were constructed using the support vector machine (SVM), random forest (RF) and back propagation neural network (BPNN) algorithms. The results showed that, compared with the “trilateral” parameter and the empirical vegetation index, the spectral index constructed by the hyperspectral reflectance after differential transformation had a stronger correlation with potato LCC_A_ and LCC_W_. Compared with no treatment, the correlation between spectral index and potato LCC and the prediction accuracy of the model showed a trend of decreasing after initial growth with the increase in differential order. The highest correlation index after 0–2 order differential treatment is DI, and the maximum correlation coefficients are 0.787, 0.798, 0.792, 0.788 and 0.756, respectively. The maximum value of the spectral index correlation coefficient after each order differential treatment corresponds to the red edge or near-infrared band. A comprehensive comparison shows that in the LCC_A_ and LCC_W_ estimation models, the RF model has the highest accuracy when combination 3 is used as the input variable. Therefore, it is more recommended to use the LCC_A_ to estimate the chlorophyll content of crop leaves in the agricultural practices of the potato industry. The results of this study can enhance the scientific understanding and accurate simulation of potato canopy spectral information, provide a theoretical basis for the remote sensing inversion of crop growth, and promote the development of modern precision agriculture.

## 1. Introduction

Potato, the fourth largest staple crop in the world, is widely distributed and exhibits strong adaptability, high yield, and rich nutritional content. It is suitable for storage as both food and industrial raw material, playing a crucial role in improving people’s living standards and ensuring food security [1]. Shaanbei, as one of the major potato-producing regions in China, possesses soil, temperature, and light conditions favorable for the growth and development of potatoes. However, outdated irrigation and fertilization techniques in this region have led to soil fertility degradation and environmental pollution, severely hindering the development of its potato industry [2]. Therefore, addressing the issues of unstable potato yields and inconsistent quality in this area is imperative. Leaf chlorophyll content (LCC) serves as a vital indicator for measuring crop growth, reflecting the growth status and health of crops. Monitoring its content changes aids in distinguishing the physiological characteristics of crops [3]. In recent years, with the rapid development of intelligent agriculture, the rapid and non-destructive estimation of chlorophyll content has been realized, which is of great significance for evaluating and managing crop canopy photosynthetic capacity.

Traditional methods for determining chlorophyll content mainly rely on ethanol extraction, which is time-consuming and cumbersome [4,5,6]. In recent years, commonly used units for chlorophyll content include chlorophyll content per unit leaf area (LCC_A_) and chlorophyll content per unit fresh weight (LCC_W_) [7]. Expressing LCC_A_ is not affected by changes in crop plant internal water content, resulting in more stable outcomes. Meanwhile, LCC_W_ is widely used in agricultural research to describe chlorophyll content [8,9]. Thus, clarifying the chlorophyll content in different measurement units is of significant importance for reflecting the actual value of crop chlorophyll. Traditional measurement methods are destructive and yield unstable results. Utilizing hyperspectral remote sensing technology provides a new approach for monitoring dynamic changes in crop leaf chlorophyll and offers technical means for selecting the most representative measurement units of crop leaf chlorophyll. Modern information technology provides a new method for intelligent agriculture. With the rapid development and integration of modern information technologies such as remote sensing, big data, machine learning and cloud computing, other technologies such as intelligent identification, accurate measurement, model construction, information collection are becoming more and more mature. It provides a new method for monitoring crop growth parameters by remote sensing, which is of great significance for crop water and fertilizer management and agricultural decision-making [10]. Liu et al. (2021) collected SPAD and remote sensing information of soybean leaves and successfully monitored the chlorophyll content using mathematical models [11]. Based on feature optimization, Zhao et al. (2022) used a variety of machine learning methods to invert farmland surface soil moisture. The experimental results show that the random forest model has higher inversion accuracy and the best fitting effect, and the inversion accuracy is greatly improved after feature optimization [12]. Existing studies mostly construct spectral indices from original canopy hyperspectral reflectance to infer crop growth physiological indicators, but the prediction accuracy and results are not satisfactory. Introducing differential transformation methods can reduce noise interference, enhance model applicability, and optimize fitting effects. Shi et al. (2023) selected the optimal spectral indices and established models using first-order differentially processed hyperspectral reflectance, which significantly improved model accuracy [13]. Zhao et al. (2022) used five methods to process the original spectrum, and found that FOD achieved good results regardless of the modeling method [14]. Currently, chlorophyll content determination often involves averaging chlorophyll content at the individual plant level [15]. Although this method is simple and easy to implement, it fails to accurately reflect the overall level of LCC. Spectral indices are linear or nonlinear combinations of different sensitive bands, closely related to the reflection, absorption, and growth of different plants in different spectral bands. Constructing a prediction model requires appropriate band combinations to enhance model accuracy [16]. When plants are subjected to disease stress, chlorophyll digestion, water content reduction and coverage reduction often accompany plant growth [17], leading to the degree of reflection of canopy spectral information on plant physiological growth indicators to decrease significantly [18]. In such cases, the use of spectral indices related to relevant bands may fail to extract all spectral information, resulting in poor model fitting [19]. A correlation matrix analysis is commonly used in crop growth parameter and spectral index correlation analysis. By selecting the optimal bands highly correlated with crop growth physiological indicators across the full spectrum, it greatly enhances the utilization of spectral information and optimizes model performance [20].

This study utilized spectral data and employed the correlation coefficient method to select three sensitive parameters for potato LCC_A_ and LCC_W_. Additionally, empirical vegetation indices, vegetation indices obtained from the differentiation of spectral bands from 0 to 2 (with a step size of 0.5), and the most highly correlated parameters among these three spectral parameters were identified. These parameters were divided into four combinations and used as inputs for model construction. Support Vector Machine (SVM), Random Forest (RF), and Back Propagation Neural Network (BPNN) were employed to build prediction models for potato LCC_A_ and LCC_W_. The study aimed to identify the most effective method for reflecting crop chlorophyll content to enhance the scientific understanding and accurate simulation of potato canopy spectral information, providing a theoretical basis for the remote sensing inversion of crop growth.

## 2. Materials and Methods

### 2.1. Research Area and Test Design

**Figure 1:**
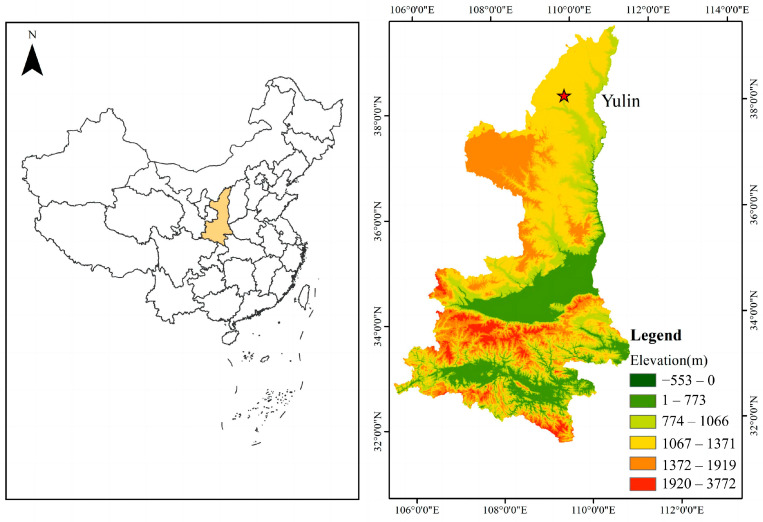
Geographic location of study area.

This experiment was conducted at the Potato Experimental Demonstration Station of Northwest A&F University in Yulin City (Figure 1), Shaanxi Province, China (38°23′ N, 109°43′ E) during the months of May to October in both 2022 and 2023. The experimental variety used was the local main cultivar, ‘Qingshu 9’. Planting took place on 5 May 2022, and 1 May 2023, respectively. In 2022, the average temperature during the entire potato growing period was 22 °C, and the total rainfall was 482.20 mm. In 2023, the average temperature during the entire potato growing period was also 22 °C, while the total rainfall was 212.10 mm. The soil was sandy loam, with the following physical and chemical properties: the bulk density of the cultivation layer (0–40 cm) was 1.73 g/cm^3^, the ammonium nitrogen content was 6.35 mg/kg, the nitrate nitrogen content was 11.45 mg/kg, the available phosphorus content was 4.43 mg/kg, the available potassium content was 107 mg/kg, the pH value was 8.1 (H_2_O was used to determine soil pH in the experiment), and the organic matter content was 4.31 g/kg. The experiment encompassed five nitrogen application levels: N0 (0 kg N/hm^2^), N1 (90 kg N/hm^2^), N2 (180 kg N/hm^2^), N3 (270 kg N/hm^2^), and N4 (360 kg N/hm^2^). Additionally, two biochar application levels were implemented: B0 (0 t/hm^2^) and B1 (30 t/hm^2^), resulting in a total of 10 experimental treatments. Phosphorus and potassium fertilizers were applied once before sowing, and nitrogen fertilizers were applied together using the water and fertilizer integration facilities during irrigation. The test fertilizers were urea (N—46%), diammonium phosphate (N—18%, P_2_O_5_—46%) and potassium nitrate (N—13.5%, K_2_O—46%). Each treatment was replicated three times, yielding a total of 30 plots. The plot dimensions were 4 m × 12 m, equivalent to 48 m^2^, and the plots were arranged randomly with a protective strip of 3 m surrounding the experimental area. The potatoes were planted by artificial sowing, with a row spacing of 0.9 m, a plant spacing of 25 cm, and a sowing depth of 8~10 cm. Before potato planting, biochar was evenly incorporated into the top 20 cm of the soil and mixed evenly, and other field treatments were consistent with the locale.

### 2.2. Data Collection and Preprocessing

#### 2.2.1. Acquisition of Spectral Data

During the tuber formation stage of the potato, spectral data were collected on days with clear weather and no cloud cover. The spectral reflectance was measured using an ASD Field-Spec 3 portable spectrometer, following the method described in reference [20]. Spectral data were collected on 7 July 2022, and 8 July 2023, between 11:00 and 13:00. There are 60 groups of samples in this study.

#### 2.2.2. Acquisition of Agronomic Parameters

The Leaf Chlorophyll Content (LCC) was determined using the 100% ethanol extraction method. Potato leaves corresponding to the hyperspectral measurement plots were collected. After removing the leaf veins, leaf disks were obtained using a hole punch method. Nine leaf disks with a diameter of 1 cm were collected and thoroughly ground. Additionally, 0.1 g of the remaining crushed leaves was weighed. A total of 10 mL of 100% ethanol was added, soaking and extracting the chlorophyll in potato leaves in a dark place at room temperature for approximately 3 days. Periodic shaking during soaking can shorten the duration, until the leaves become colorless or white. After all the chlorophyll in the crushed leaves was extracted into the ethanol solution (adjusted to a total volume of 25 mL), the absorbance at wavelengths of 663 nm and 645 nm was measured. The LCC_A_ and LCC_W_ were calculated using the following formulas [8,9]:

(1) $$\mathrm{Chlorophyll}\;a\;\mathrm{content}=(12.7D_{663\mathrm{nm}}-2.69D_{645\mathrm{nm}})\times \frac{1}{40\times m}$$

(2) $$\mathrm{Chlorophyll}\;b\;\mathrm{content}=(22.9D_{663\mathrm{nm}}-4.68D_{645\mathrm{nm}})\times \frac{1}{40\times m}$$

(3) $$\mathrm{Total}\;\mathrm{chlorophyll}\;\mathrm{content}=(20.21D_{645\mathrm{nm}}+8.02D_{663\mathrm{nm}})\times \frac{1}{40\times m}$$

In the equations, *D*_663nm_ and *D*_645nm_ represent the absorbance at 663 nm and 645 nm, respectively. *m* denotes the fresh weight (g) or leaf area (dm^2^). When *m* represents the fresh weight of the leaf, it yields the LCC_W_. When *m* represents the leaf area, it yields the LCC_A_.

Specific Leaf Weight (SLW) refers to the weight of leaf per unit leaf area (fresh weight). In this study, Specific Leaf Weight (g/dm^2^) is calculated as the LCC_A_ divided by the LCC_W_.

#### 2.2.3. Spectral Data Processing

The original spectra of 60 samples in this study were obtained using View Spec Pro Version 6.2 software. In this study, 0–2 order fractional differential (FD) processing was performed on the spectral data after SG (Savitzky–Golay) smoothing pretreatment [20,21]. SG smoothing was implemented in The Unscrambler X 10.4 software.

The preprocessing of spectral data and the calculation of vegetation indices were conducted using MATLAB 2022 (MathWorks, Inc., Natick, MA, USA). The drawing charts were created using Origin 2024 (OriginLab Corp., Northampton, MA, USA).

### 2.3. Model Construction and Validation

Three different spectral indices were selected to more accurately screen for the wavelength combinations with the highest correlation with LCC_A_ and LCC_W_:

(1) Previous research has demonstrated better correlations between empirical vegetation indices and crop parameters; therefore, this study also selected some empirical vegetation indices.
(2) The “trilateral” spectral parameters, which encompass the regions in the blue edge, yellow edge, and red edge spectra, are derived by extracting the peak value, valley value, area, or a combination of different bands from the blue edge, yellow edge, and red edge.
(3) The inversion of agricultural parameters can be effectively achieved by selecting any two-band vegetation index as the input parameter for the model. In this study, three arbitrary dual-band indices were initially chosen and then subjected to a 0–2 order fractional differential operation. Within the range of its spectral measurement wavelength, the combination index of the optimal order and the best vegetation index were selected.

Then, the two spectral indices with the highest correlation to potato LCC_A_ or LCC_W_ were further selected, constituting the optimal combination indices. The detailed calculation formulas are provided in Table 1.

### 2.4. Model Approach

From the empirical spectral indices, “trilateral” spectral parameters, fractional order differentiation processed spectral indices within 0–2 order, and all spectral indices, the spectral index with the best correlation with LCC_A_ and LCC_W_ was selected as the model input. Subsequently, SVM, RF and BPNN models were separately employed to model LCC_A_ and LCC_W_. For SVM, both Gaussian kernel and polynomial kernel were used as base kernel functions. The model parameters C and γ are 20 and 0.02, respectively [30]. RF belongs to the bagging algorithm in Ensemble Learning. The CART tree model is used as the base learner, the number of decision trees is 100 [31]. In BPNN, through data forward propagation and error back propagation, the input has undergone multiple iterations and repeated training [32]. The final fitted result is the average of multiple predictions from the machine learning model.

### 2.5. Model Evaluation Index

The model fitting results are evaluated using R^2^, RMSE, and MRE. A higher R^2^ signifies improved predictive accuracy, whereas smaller RMSE and MRE values indicate greater model stability and more focused prediction outcomes [21].

## 3. Results

### 3.1. LCC_A_, LCC_W_, SLW and Yield (GY)

**Figure 2:**
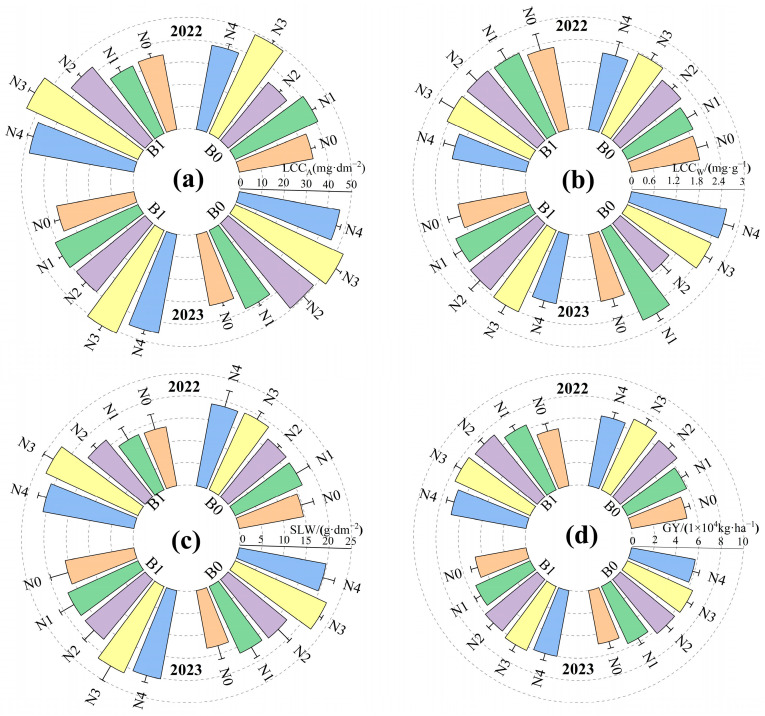
LCC_A_ (**a**), LCC_W_ (**b**), SLW (**c**), and GY (**d**) under different treatments.

In Figure 2, the trends of LCC_A_, LCC_W_, SLW, and GY under different treatments are illustrated. When the application rate of biochar is constant, LCC_A_, LCC_W_, SLW, and GY initially increase and then decrease with the increase in nitrogen fertilizer. Among them, the highest values of LCC_A_, LCC_W_, SLW, and GY are observed at N3. When the nitrogen fertilizer application rate is constant, the values of LCC_A_, LCC_W_, SLW, and GY in treatment B1 are higher than those in treatment B0, with increases of 5.44%, 7.61%, 1.32%, and 4.82%, respectively, compared to B0. Treatment B1N3 maximally enhances LCC_A_, LCC_W_, SLW, and GY of the crops.

The significant analysis of the effects of different biochar types and nitrogen application rates on LCC_A_, LCC_W_, SLW and yield (GY) is presented in Table 2. Different nitrogen fertilizer application rates significantly affect LCC_A_, SLW, and GY (*p* < 0.05). Different biochar application rates significantly affect LCC_A_ and GY, and the interaction between nitrogen fertilizer and biochar application rates significantly influences LCC_A_, SLW, and GY (*p* < 0.05). The effects of nitrogen fertilizer application rates, biochar application rates, and their interaction on LCC_W_ are not significant.

### 3.2. Correlation Analysis between LCC_A_, LCC_W_ and Spectral Index

**Figure 3:**
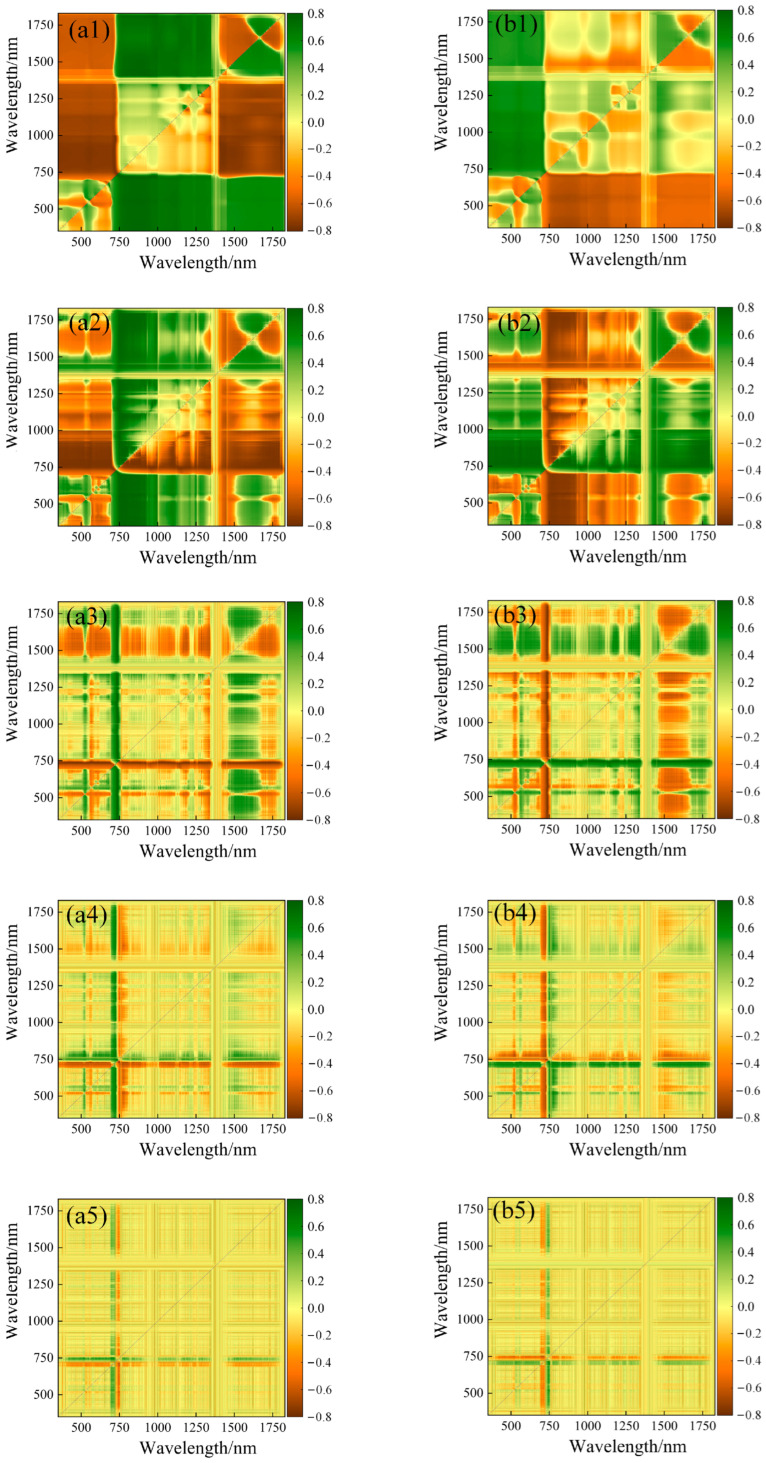
Correlation matrix of DI, SAVI with LCC_A_ (**a1**–**a5**,**b1**–**b5**).

**Figure 4:**
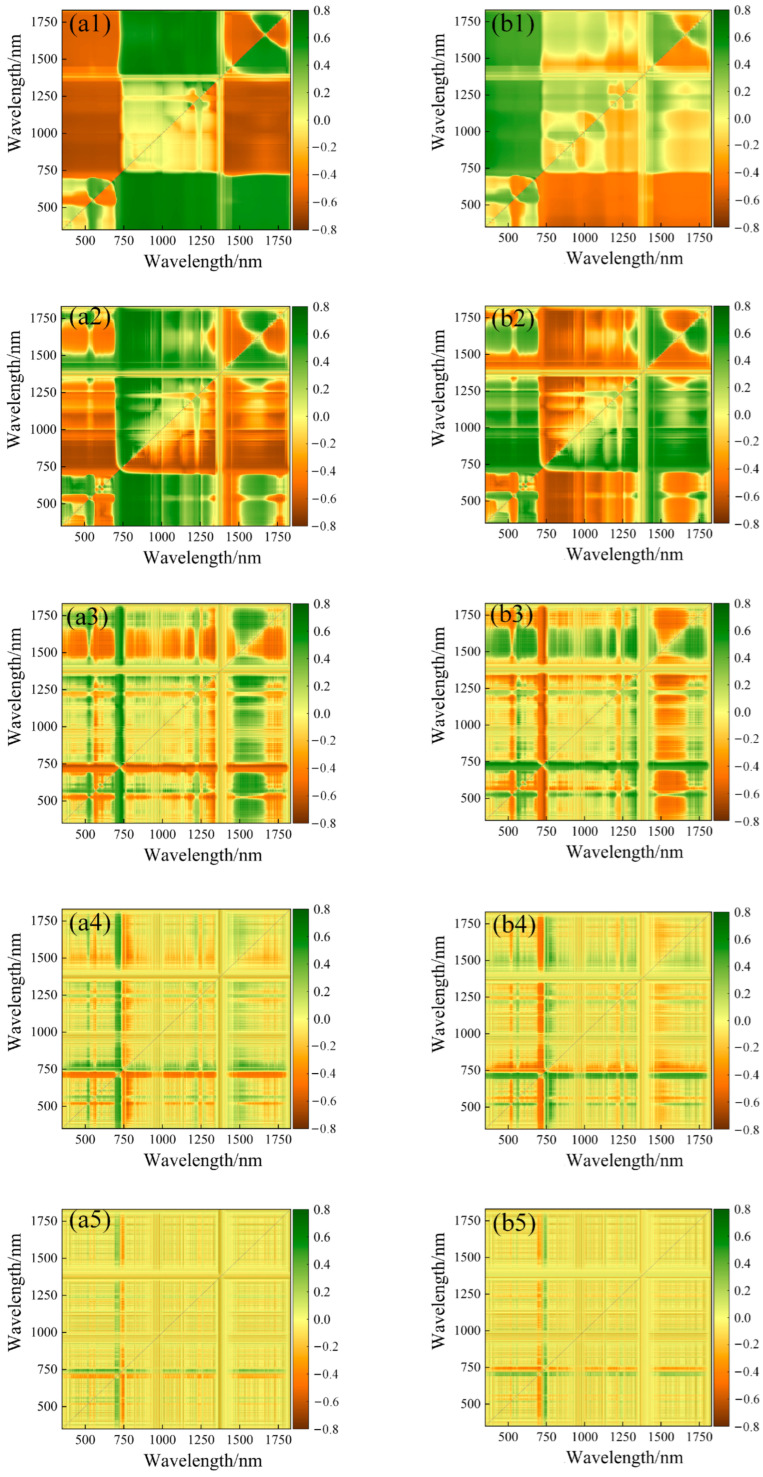
Correlation matrix of DI, SAVI with LCC_W_ (**a1**–**a5**,**b1**–**b5**).

The correlation analysis between various spectral indices and potato LCC_A_ and LCC_W_ was conducted to select the optimal vegetation index as the model input variable. Table 3 displays the correlation coefficients between empirical spectral indices, “trilateral” parameters, and potato LCC_A_ and LCC_W_. The correlation analysis between empirical spectral indices and potato LCC_A_ indicates that the top seven indices with the highest correlation coefficients are IPVI, SR1, SR705, SR3, SR680, GRVI, and CARI, ranging from 0.4 to 0.8. Among them, IPVI exhibits the highest correlation coefficient of 0.771. In contrast, the top seven indices with the optimal correlation between empirical spectral indices and potato LCC_W_ are IPVI, CARI, SR1, SR3, SR705, SR680, and SIPI, ranging from 0.3 to 0.7. The Integrated Phenotypic Vegetation Index (IPVI) exhibits a peak correlation of 0.695. When assessed against LCC_W_, empirical spectral indices have demonstrated a higher degree of correlation with LCC_A_. Furthermore, the so-called “trilateral” parameters have shown a consistently strong correlation with potato LCC_A_ and LCC_W_. The top seven parameters with the optimal correlation with potato LCC_A_ are SDr-SDb, SDr, Dr, Dy, Db, SDb, and Rg, ranging from 0.5 to 0.8. Among them, SDr-SDb exhibits the optimal correlation of 0.717. Similarly, the top seven parameters with the optimal correlation with potato LCC_W_ are SDr, SDr-SDb, Dr, Dy, Db, Rg, and SDb, ranging from 0.4 to 0.7. SDr shows the highest correlation coefficient of 0.613. Two arbitrary two-band spectral indices were constructed based on the spectral reflectance after 0–2 order (step size 0.5) differential processing, and their correlation with LCC_A_ and LCC_W_ were analyzed (Table 4). A graphical representation of the correlation matrix, referred to as Figure 3 and Figure 4, was constructed. In this visualization, a color gradient ranging from yellow to green is utilized to depict the degree of correlation between various two-band spectral indices and the concentration of LCC_A_ or LCC_W_. The gradient indicates a spectrum of correlation values, transitioning from strongly negative to strongly positive. The correlation analysis between spectral indices and LCC_A_ indicates that the spectral indices constructed from spectra processed with 0.5, 1, and 1.5 order differentials exhibit significantly improved correlation coefficients with potato LCC_A_, with the highest correlation coefficient observed for DI constructed from spectra processed with a 0.5 order differential, reaching a maximum value of 0.798, with corresponding wavelength positions at (755,697). In contrast, the spectral indices constructed from spectra processed with a 2 order differential show a decrease in correlation coefficients. The order of correlation coefficients in terms of order is: 0.5 order > 1 order > 1.5 order > 0 order > 2 order. Similarly, the spectral index with the optimal correlation in the correlation analysis between spectral indices and LCC_W_ is DI processed with a 1.5 order differential, with a value of 0.737 and corresponding wavelength combination of (726,680). The order of correlation coefficients in terms of order is: 1.5 order > 1 order > 0.5 order > 2 order > 0 order. Compared to spectral indices established from the original reflectance, the correlation coefficients of spectral indices calculated from fractional order differentials significantly improved with LCC_A_ or LCC_W_.

### 3.3. Establishment of Estimation Model of LCC_A_ and LCC_W_ Based on Optimal Spectral Index

**Figure 5:**
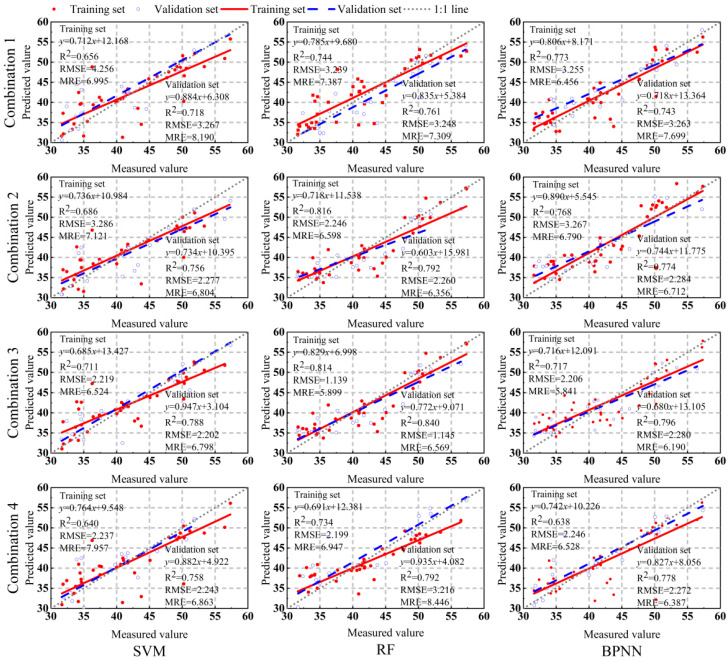
Precision evaluation of potato LCC_A_ model under different input variables and different model combinations.

**Figure 6:**
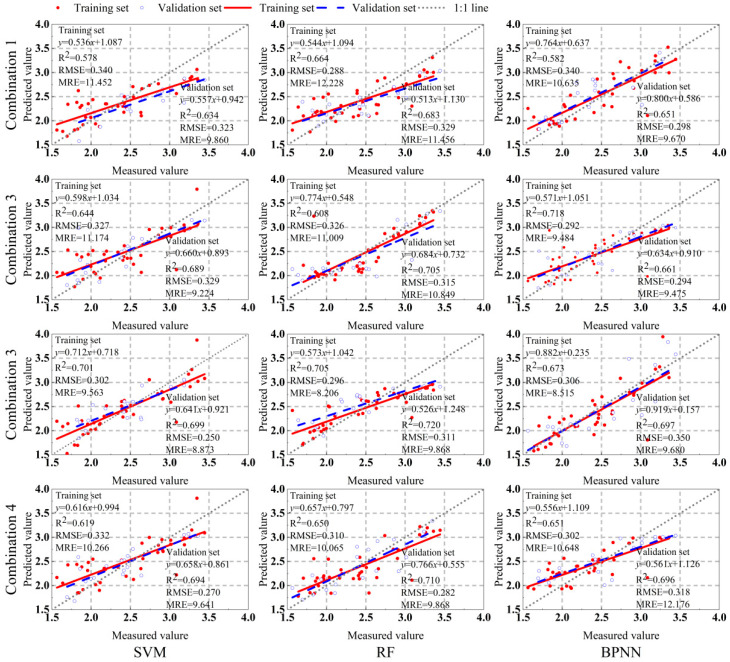
Precision evaluation of potato LCC_W_ model under different input variables and different model combinations.

Section 2.3 introduces empirical spectral indices, “trilateral” spectral parameters, and arbitrary two-band vegetation indices. The parameters with the optimal correlation in these three types of spectral parameters are divided into four combinations for correlation analysis. Then, the top seven spectral indices with the optimal correlation with potato LCC_A_ or LCC_W_ in each combination are chosen as the input for the model. Potato LCC_A_ or LCC_W_ is used as the response variable, and SVM, RF, and BPNN are used to construct prediction models for potato tuber formation period LCC_A_ and LCC_W_. The performance and fitting effect of the models are comprehensively evaluated based on three indicators: R^2^, RMSE, and MRE (Figure 5 and Figure 6).

In a parallel comparison of the three models, the model accuracy for estimating potato LCC_A_ and LCC_W_ is as follows: RF > BPNN > SVM. In the potato LCC_A_ estimation model, both RF and BPNN have R^2^ values higher than 0.7, with RMSE and MRE maintained at relatively low levels, indicating good model performance and fitting effect. In the potato LCC_A_ estimation model, when the input variables are different combinations, the validation set R^2^ values are all higher than 0.7, indicating good model performance and fitting effect. In contrast, in the potato LCC_W_ estimation model, the SVM and BPNN models have R^2^ values ranging from 0.5 to 0.7 when the input variables are combination 1, indicating a lower fitting accuracy. However, for combination 3, both the modeling set and validation set have the highest R^2^ values, with lower RMSE and MRE, specifically showing: combination 3 > combination 4 > combination 2 > combination 1. Overall, the R^2^ of the LCC_A_ model is higher than that of the LCC_W_ model, and the MRE shows lower values, indicating higher model accuracy and better performance and fitting effect. When the input variables and modeling methods are combination 3 and RF, the optimal potato LCC_A_ and LCC_W_ prediction models can be constructed. The R^2^ values of the validation set are 0.840 and 0.720, RMSE values are 1.145 and 0.311, and MRE values are 6.569% and 11.868%, respectively.

## 4. Discussion

In recent years, with the rapid development and integration of modern information technologies such as remote sensing, big data, machine learning and cloud computing, there have been numerous applications in monitoring crop growth or pest and disease infestations in agriculture. Hyperspectral imaging, due to its wide spectral range and nearly continuous spectral information of objects, can accurately record multidimensional information and component data [33]. It has been widely used in monitoring crop parameters such as leaf area index (LAI) [21], LCC [13], above-ground biomass (AGB) [34], soil moisture content [35], and surface parameters. Chlorophyll content directly determines the photosynthetic activity of crops and is an important physiological indicator for evaluating crop growth status. Combining hyperspectral remote sensing to estimate crop LCC is beneficial for accurately assessing its estimation capability and comprehensively evaluating crop growth status [36].

The construction of three types of spectral indices or parameters, including empirical spectral indices, “trilateral” spectral parameters, and arbitrary two-band spectral indices, revealed that the selection of arbitrary two-band spectral indices showed the highest correlation with potato LCC. This is because the arbitrary two-band spectral indices created by combining two bands utilize hyperspectral reflectance data processed through 0–2 order differentials, which helps reduce the basic background noise of the original spectral reflectance data and highlights their detailed spectral features [37]. As the order of differentiation increases, both the correlation between spectral indices and potato LCC and the predictive fitting performance of the model initially increase, but then decrease. When fractional order differentials (such as 0.5 order and 1.5 order) are used, the correlation between arbitrary two-band spectral indices and potato LCC exceeds that of the integer-order differentials (such as 1 order and 2 order). This is mainly because fractional order differentials can capture gradient information missed by integer-order differentials [38]. Most empirical vegetation indices based on fixed bands tend to saturate. When the crop canopy coverage is high, empirical vegetation indices tend to saturate, leading to decreased sensitivity to the reflecting of chlorophyll content and thus a decrease in correlation [39]. In sparse canopy conditions, where soil reflectance dominates, the effectiveness of empirical vegetation indices in reflecting vegetation growth parameters is often poor [40]. Additionally, due to the influence of the crop growth stage, environment, and pests and diseases, different spectral information may be generated, resulting in the phenomenon of “same object with different spectra” or “different objects with the same spectrum”. In such cases, the use of empirical vegetation indices and “trilateral” parameters based on correlated bands may not fully utilize spectral information, leading to reduced correlation [41].

Our study utilized the rich spectral information contained in hyperspectral data to construct various spectral indices combined with different machine learning methods. We aimed to establish models for predicting LCC_A_ and LCC_W_ in potatoes, and to explore the rationality and applicability of these two different units of chlorophyll content. The results indicated that the LCC_A_ model exhibited higher R^2^ compared to LCC_W_, with a lower Mean Relative Error (MRE), indicating higher accuracy and better fitting of the model. This suggests that using hyperspectral data to extract information about crop LCC_A_ is richer and more correlated compared to LCC_W_. This is attributed to the instability of chlorophyll content represented per unit fresh weight under different water and fertilizer supply conditions and growth environments. When chlorophyll content is expressed per unit leaf area, its representation per unit fresh weight is greatly affected by leaf water content variations, resulting in significant variability [42]. Therefore, expressing LCC_A_ can effectively avoid the interference of crop leaf water content, better approximate the true value of crop leaf chlorophyll content, and make full use of spectral information to accurately monitor crop photosynthetic capacity and understand crop growth conditions in a timely manner. Among the three models used in this study, the LCC_A_ and LCC_W_ prediction models-based RF method showed the optimal fitting performance, attributed to RF’s good noise and interference resistance and its resistance to overfitting [43], whereas Back Propagation Neural Network (BPNN) suffers from slow convergence and is prone to local minima during training [44]. Support Vector Machine (SVM) exhibited the lowest accuracy, possibly due to its sensitivity to model parameters such as the kernel function and penalty factor, which hindered its predictive ability [45]. Therefore, the RF model is considered the optimal method for predicting crop LCC, and expressing LCC_A_ is recommended for estimating crop leaf chlorophyll content in the agricultural practices of the potato industry.

This study mainly focuses on the model inversion results of LCC_W_ and LCC_A_, which shows that LCC_A_ has a strong ability to represent the real chlorophyll content of crops. These strategies can efficiently and non-destructively monitor the chlorophyll content of crops, grasp the real-time growth status of crops, and formulate corresponding solutions. Under the premise of paying attention to the efficient and rational use of water resources and fertilizers, they can effectively guide precision fertilization, scientific irrigation, and integrated pest control. These models not only save agricultural water and fertilizer, but also make an important contribution to the sustainable utilization of agricultural resources and the protection of the ecological environment. In the future, multi-source remote sensing data (hyperspectral, multispectral, thermal infrared, etc.) will be used as model input variables, and other types of models will be tried. The field measured data (different varieties, regions, time and space) will be compared and verified at a larger scale, in order to strengthen the real-time monitoring of crop physiological growth and promote the development of intelligent agriculture.

## 5. Conclusions

In this study, four combinations of model input variables were constructed, including empirical spectral indices, “trilateral” spectral parameters, any two-band vegetation indices, and the most highly correlated parameters among these three spectral parameters. SVM, RF and BPNN machine learning methods were employed to construct models for predicting LCC_A_ and LCC_W_ during the tuber differentiation stage of potatoes. The conclusions drawn from the study are as follows:

(1) Compared to “trilateral” parameters and empirical vegetation indices, any two-band vegetation indices constructed from hyperspectral reflectance after fractional order differentiation processing exhibit stronger correlations with potato LCC. As the order of differentiation increases, both the correlation between spectral indices and potato LCC and the predictive accuracy of the models initially increase but then decrease. When employing fractional order differentiations (e.g., 0.5th order and 1.5th order), the correlation between any two-band spectral indices and potato LCC exceeds that obtained when using integer-order differentiations (e.g., 1st order and 2nd order). Among them, the maximum correlation coefficients of the DI with the highest correlation after 0–2 order differentiation processing are: 0.787, 0.798, 0.792, 0.788, and 0.756, respectively.
(2) In the constructed LCC_A_ and LCC_W_ models, the performance and fitting effects are as follows: RF > BPNN > SVM, with the input combinations ranked as follows: combination 3 > combination 4 > combination 2 > combination 1. The RF method consistently demonstrates the highest accuracy and best fitting performance in model construction. The optimal input variables and modeling method for both LCC_A_ and LCC_W_ models are combination 3 and RF method. Therefore, expressing LCC_A_ is recommended for estimating crop leaf chlorophyll content in agricultural practice.

## Figures and Tables

**Table 1:** The empirical vegetation index selected for the study.

Selected Spectra Parameters	Calculation Formula	Reference
CARI	(R_700_ − R_670_) − 0.2 × (R_700_ + R_670_)	[22]
GRVI	R_800_/R_550_	[22]
PRI	(R_570_ − R_530_)/(R_570_ + R_530_)	[22]
IPVI	R_800_ × (R_800_ + R_670_)	[22]
PRI1	(R_531_ − R_570_)/(R_531_ + R_570_)	[23]
SR1	R_750_/R_700_	[24]
SR3	R_750_/R_550_	[24]
SR705	R_750_/R_705_	[25]
SR680	R_800_/R_680_	[25]
SIPI	(R_800_ − R_445_)/(R_800_ − R_680_)	[25]
D_b_	The highest value of the blue edge band (490–530 nm) in the 1-FD order spectral.	[26]
Dy	The highest value of the yellow edge band (462–642 nm) after the 1-FD order treatment.	[26]
Dr	The highest value of the red edge band (670–760 nm) after the 1-FD order treatment.	[27]
R_g_	The highest value of the green edge band (510~560 nm).	[27]
R_r_	The lowest value of the red edge band (650~690 nm).	[27]
SD_b_	The sum of the blue edge wavelength range in the spectral reflectance after the 1-FD order treatment.	[28]
SD_y_	The sum of the yellow edge wavelength range after the 1-FD order treatment	[28]
SD_r_	The sum of the red edge wavelength range after the 1-FD order treatment.	[28]
SDr-SDb	/	[29]
SDr/SDy	/	[29]
Difference Index (DI)	$R_{i}$ − $R_{j}$	[13]
Soil-Adjusted Vegetation Index (SAVI)	$\left(1+0.16\right)\frac{R_{i}-R_{j}}{R_{i}+R_{j}+0.16}$	[13]

Notes: $R_{i}$ (*i* = 1, 2, 3) is any value of the wavelength reflectivity in the measurement range (350~1830 nm), 1-FD is the first-order differential, and *R*_number_ is the spectral reflectivity of the digital band.

**Table 2:** Effects of different biochar and nitrogen application rates on LCC_A_, LCC_W_, SLW and GY.

Year	Treatment		LCC_A_	LCC_W_	SLW	GY
			mg·dm^−2^	mg·g^−1^	g·dm^−2^	kg·ha^−1^
2022	B0	N0	33.60 hi	2.07 ab	16.54 bcd	50,520.34 f
		N1	33.87 hi	2.16 ab	19.00 abcd	58,533.81 e
		N2	38.44 fgh	2.34 ab	14.63 cd	65,618.02 d
		N3	49.00 bcd	2.64 ab	18.72 bcd	69,750.80 bc
		N4	40.78 ef	2.33 ab	16.80 bcd	63,574.08 d
	B1	N0	33.55 hi	2.24 ab	13.43 cd	52,084.25 f
		N1	34.09 hi	2.65 ab	13.65 cd	64,221.69 d
		N2	42.74 ef	2.70 ab	16.53 bcd	71,766.71 ab
		N3	54.95 cde	2.73 ab	22.73 ab	74,203.79 a
		N4	44.77 a	2.50 ab	20.73 abcd	68,307.19 c
2023	B0	N0	35.22 ghi	2.07 ab	15.66 bcd	46,397.08 e
		N1	39.75 fg	2.45 ab	17.32 bcd	53,743.41 d
		N2	40.87 ef	2.57 ab	17.33 bcd	60,027.95 b
		N3	50.91 abc	2.69 ab	21.41 abc	63,212.80 a
		N4	45.11 de	2.11 ab	20.17 abcd	60,036.28 b
	B1	N0	32.56 i	2.04 ab	13.14 d	48,711.15 e
		N1	38.81 fgh	2.89 a	16.48 bcd	56,542.28 c
		N2	49.38 bcd	1.88 b	17.01 bcd	62,935.39 a
		N3	53.35 ab	2.97 a	26.52 a	64,432.88 a
		N4	45.79 cde	2.76 ab	19.74 abcd	58,184.26 bc
Significant level						
	B		**	ns	ns	**
	N		**	ns	**	**
	B×N		**	ns	*	*

Notes: The letters after the values of each column indicated that there were significant differences between treatments (*p* < 0.05), and * (*p* < 0.05) and ** (*p* < 0.01) indicated that there were significant differences in different degrees, ns means no significant difference.

**Table 3:** Empirical spectral index and ‘trilateral’ parameters and potato LCC_A_ and LCC_W_ correlation coefficients.

Index	Spectral Index Category	Spectral Index	r
LCC_A_	Empirical spectral index	CARI	0.496
		GRVI	0.404
		PRI	0.317
		IPVI	0.771
		PRI1	0.338
		SR1	0.669
		SR3	0.533
		SR705	0.658
		SR680	0.504
		SIPI	0.372
		Db	0.567
	“trilateral” parameters	Dy	0.568
		Dr	0.673
		Rg	0.536
		Rr	−0.087
		SDb	0.565
		SDy	−0.262
		SDr	0.711
		SDr-SDb	0.717
		SDr/SDy	0.432
LCC_W_	Empirical spectral index	CARI	0.563
		GRVI	0.133
		PRI	−0.106
		IPVI	0.695
		PRI1	0.123
		SR1	0.515
		SR3	0.473
		SR705	0.394
		SR680	0.383
		SIPI	0.302
		Db	0.531
	“trilateral” parameters	Dy	0.532
		Dr	0.560
		Rg	0.548
		Rr	0.106
		SDb	0.481
		SDy	−0.064
		SDr	0.613
		SDr-SDb	0.612
		SDr/SDy	0.398

**Table 4:** Optimal spectral index wavelength combinations under different differential orders.

Index	Spectral Index	Differential Order	r_max_	Position of Wavelength (i, j)/(nm)
LCC_A_	DI	0	0.787	740,733
		0.5	0.798	755,697
		1	0.792	737,758
		1.5	0.788	736,748
		2	0.756	702,753
	SAVI	0	0.700	708,756
		0.5	0.787	694,755
		1	0.792	754,745
		1.5	0.785	748,736
		2	0.756	753,702
LCC_W_	DI	0	0.684	757,724
		0.5	0.723	756,671
		1	0.723	739,670
		1.5	0.737	726,680
		2	0.702	694,751
	SAVI	0	0.612	674,678
		0.5	0.706	671,756
		1	0.723	670,739
		1.5	0.736	751,731
		2	0.702	751,694

## Data Availability

Data are contained within the article.
